# Impaired NK Cell Responses to Pertussis and H1N1 Influenza Vaccine Antigens in Human Cytomegalovirus-Infected Individuals

**DOI:** 10.4049/jimmunol.1403080

**Published:** 2015-04-08

**Authors:** Carolyn M. Nielsen, Matthew J. White, Christian Bottomley, Chiara Lusa, Ana Rodríguez-Galán, Scarlett E. G. Turner, Martin R. Goodier, Eleanor M. Riley

**Affiliations:** * Department of Immunology and Infection, London School of Hygiene and Tropical Medicine, London WC1E 7HT, United Kingdom; and; † Department of Infectious Disease Epidemiology, London School of Hygiene and Tropical Medicine, London WC1E 7HT, United Kingdom

## Abstract

NK cells contribute to postvaccination immune responses after activation by IL-2 from Ag-specific memory T cells or by cross-linking of the low-affinity IgG receptor, CD16, by Ag–Ab immune complexes. Sensitivity of NK cells to these signals from the adaptive immune system is heterogeneous and influenced by their stage of differentiation. CD56^dim^CD57^+^ NK cells are less responsive to IL-2 and produce less IFN-γ in response to T cell–mediated activation than do CD56^bright^ or CD56^dim^CD57^−^ NK cells. Conversely, NK cell cytotoxicity, as measured by degranulation, is maintained across the CD56^dim^ subsets. Human CMV (HCMV), a highly prevalent herpes virus causing lifelong, usually latent, infections, drives the expansion of the CD56^dim^CD57^+^NKG2C^+^ NK cell population, skewing the NK cell repertoire in favor of cytotoxic responses at the expense of cytokine-driven responses. We hypothesized, therefore, that HCMV seropositivity would be associated with altered NK cell responses to vaccine Ags. In a cross-sectional study of 152 U.K. adults, with HCMV seroprevalence rate of 36%, we find that HCMV seropositivity is associated with lower NK cell IFN-γ production and degranulation after in vitro restimulation with pertussis or H1N1 influenza vaccine Ags. Higher expression of CD57/NKG2C and lower expression of IL-18Rα on NK cells from HCMV seropositive subjects do not fully explain these impaired responses, which are likely the result of multiple receptor–ligand interactions. This study demonstrates for the first time, to our knowledge, that HCMV serostatus influences NK cell contributions to adaptive immunity and raises important questions regarding the impact of HCMV infection on vaccine efficacy.

## Introduction

Natural killer cells are traditionally classified as cells of the innate immune system but can also act as mediators of adaptive immunity. In addition to their well-recognized role in Ab-dependent cytotoxicity (ADCC), recent research has demonstrated a potential contribution to adaptive responses through their activation by Ag-specific CD4^+^ T cell–derived IL-2 (1–7). The heightened IFN-γ response of NK cells in the context of a vaccine recall response suggests that NK cells may play a role in protection from vaccine-preventable diseases, particularly as NK cells respond more quickly than T cells and comprise as much as 70% of all IFN-γ–producing cells in the first 12–24 h of the recall response (3).

We have shown, using the individual components of the diphtheria toxoid/tetanus toxoid/whole-cell pertussis vaccine, that activation of NK cells after restimulation with vaccine Ags is heterogeneous, with CD56^bright^ and CD56^dim^CD57^−^ NK cells being most responsive as measured by surface expression of the high-affinity IL-2 receptor (CD25) and accumulation of intracellular IFN-γ (CD25^+^IFN-γ^+^) (6). Expression of CD57 by CD56^dim^ NK cells was associated with a reduced capacity to produce IFN-γ, although degranulation responses were maintained (6). These data are consistent with the accepted model of NK cell maturation whereby acquisition of CD57 is a marker of decreased sensitivity to exogenous cytokine stimulation (8, 9).

Human CMV (HCMV) infection drives profound changes in the NK cell repertoire. In particular, HCMV infection is strongly associated with preferential expansion of the CD56^dim^CD57^+^NKG2C^+^ NK cell subset (10–12). This has direct implications for NK cell function as CD56^dim^CD57^+^NKG2C^+^ NK cells degranulate and secrete cytokines such as IFN-γ and TNF-α in response to cross-linking of CD16 (by IgG) or natural cytotoxicity receptors (by infected, stressed, or transformed cells) but respond poorly to proinflammatory cytokines such as IL-12 and IL-18 (12, 13).

These observations imply that, in the context of infection or vaccination, NK cells from HCMV-seropositive (HCMV^+^) individuals may effectively mediate ADCC after cross-linking of CD16 by IgG in immune complexes (11, 13, 14), but may respond poorly to inflammatory cytokines (reviewed in Ref. 15). Specifically, the expanded CD56^dim^CD57^+^NKG2C^+^ NK cell subset may be less sensitive to IL-2 produced by Ag-specific CD4^+^ T cells and IL-12/IL-18 from accessory cells, such as dendritic cells and macrophages (3, 6). However, much of the data on skewing of the NK cell repertoire in HCMV^+^ individuals comes from studies of hematopoietic stem cell or solid organ transplantation (11, 16, 17), and follow-up of these patients over time, in terms of susceptibility to infection or response to vaccination, is lacking. As a result, the true functional significance of HCMV-driven NK cell phenotypic changes is poorly understood. Moreover, previous investigations of the impact of HCMV infection on vaccination have produced rather inconsistent results, with some studies reporting impaired vaccine responses in HCMV^+^ donors (18–23), whereas others find no impact of HCMV infection (24–27). The impact of HCMV-driven immune differentiation on vaccine responsiveness and efficacy is therefore still unclear.

The aim of this study, therefore, is to compare NK cell responses to Ags previously encountered during immunization (*Bordetella pertussis*) or during natural infection (H1N1 influenza virus) in HCMV^−^ and HCMV^+^ individuals.

## Materials and Methods

### Study subjects

Volunteers (*n* = 152) were recruited from staff and students at the London School of Hygiene and Tropical Medicine. All subjects gave written consent and the study was approved by the London School of Hygiene and Tropical Medicine Ethics Committee. Each subject provided a 50-ml venous blood sample, and reported vaccination history was recorded. Subject characteristics are summarized in Table I.

### Ab detection by ELISA

Plasma was collected from heparinized whole blood and stored at −80°C until use. HCMV infection status was determined by HCMV IgG ELISA (BioKit). IgG Abs to pertussis toxin (PT; NIBSC) and to formalin-inactivated whole H1N1 influenza virus (influenza A/California/7/2006(H1N1)v(NYMC-X179A); H1N1; NIBSC) were determined using in-house ELISA assays with goat anti-human IgG-peroxidase (Sigma-Aldrich) as the secondary Ab and SIGMAFAST OPD (Sigma-Aldrich) as the substrate. IgG concentrations were calculated by interpolation from a standard curve, which was produced using anti-pertussis reference serum (NIBSC; IU/ml) or using plasma from a donor with high titers of Abs to H1N1 influenza (IgG concentration expressed in arbitrary ELISA units [AEU]) (28). The pooled AB plasma used for in vitro assays contained 6.8 IU/ml IgG to PT and had an H1N1 IgG titer of 273.8 AEU.

### PBMC preparation and culture

PBMCs were isolated from heparinized venous blood on a Ficoll–Hypaque gradient and cryopreserved in liquid nitrogen. Before use, PBMCs were thawed into complete medium (RPMI 1640 supplemented with 100 U/ml penicillin/streptomycin and 20 mM l-glutamine [Life Technologies, Lifesciences] and 10% pooled human AB plasma), washed, and rested for 30 min before use. For some experiments, AB plasma was IgG-depleted using a protein G-Sepharose column (GE Life Sciences).

PBMCs were cultured for 18 h at 37°C at 2 × 10^5^/well in 96-well U-bottom plates (Nunc) in complete medium with or without low concentration of cytokines (LCC; 12.5 pg/ml rhIL-12 [PeproTech] plus 10 ng/ml rhIL-18 [MBL, Woburn, MA]); high concentration of cytokines (HCC; 5 ng/ml rhIL-12 plus 50 ng/ml rhIL-18); rat anti-IL-2 (3 μg/ml; BD Biosciences); rat IgG2A isotype control (3 μg/ml; BD Biosciences; this was included in wells with medium alone, as well as Ag alone); 1 μg/ml formalin-inactivated whole H1N1 influenza virus (NIBSC, as described earlier); 1 IU/ml killed whole-cell *B. pertussis* (pertussis; NIBSC); or MHC class I–deficient K562 target cells (E:T ratio 2:1). GolgiStop (containing Monensin, 1/1500 concentration; BD Biosciences) and GolgiPlug (containing brefeldin A, 1/1000 final concentration; BD Biosciences) were added after 15 h. Anti-CD107a Ab (A488-conjugated; BD Biosciences) was included in the medium for the entirety of cell culture.

For activation via CD16 cross-linking, 96-well flat-bottom plates (Nunc) were coated with anti-human CD16 (BD Biosciences) or an isotype-matched control Ab (mIgG1k; BD Biosciences) overnight at 4°C. Wells were rinsed with PBS before addition of 2 × 10^5^ PBMCs/well, which had been incubated overnight at 37°C with 50 IU/ml IL-2 (PeproTech). Anti–CD107a-A488 Ab was added at the beginning of culture, and cells were harvested after 5 h.

### Flow cytometry

PBMCs were stained in 96-well U-bottom plates as described previously (6). In brief, cells were stained with fluorophore-labeled Abs to cell-surface markers, fixed, permeabilized (Cytofix/Cytoperm; BD Biosciences), and stained for intracellular molecules. The following mAbs were used: anti–CD3-V500, anti–CD56-PECy7, anti–IFN-γ–allophycocyanin, anti–CD107a-A488, anti–CD16-allophycocyanin-H7, anti–CD25-allophycocyanin-H7 (all BD Biosciences), anti–CD57-e450, anti–CD25-PerCPCy5.5, anti–CD16-allophycocyanin, anti–CD25-PE, anti–IL-18Rα–PE, anti–IL-18Rα–FITC, anti–IFN-γ–allophycocyanin-e780, anti–CD16–allophycocyanin-e780 (all e-Biosciences), anti–NKG2C-allophycocyanin, anti–NKG2C-PE (both R&D Systems), and anti–NKG2A-FITC (Miltenyi). IL-12Rβ2 Ab was conjugated using EasyLink PE-Cy5 (Abcam). Cells were acquired on an LSRII flow cytometer (BD Biosciences) using FACSDiva software. Data analysis was performed using FlowJo V10 (Tree Star). FACS gates set on unstimulated cells (medium alone or isotype controls) were applied in standard format across all samples and all conditions.

### NKG2C genotyping

DNA was extracted from whole blood using a Wizard genomic DNA extraction kit (Promega). Donors were then genotyped for *NKG2C* using touch-down PCR (Phusion High Fidelity PCR kits; New England Biolabs) as described previously (29, 30).

### Statistical analyses

Statistical analysis of flow cytometry data was performed using Prism 6 (GraphPad), or STATA/IC 13 (StataCorp), as detailed in the figure legends. Responses where the gated cell subset contained <100 cells were excluded. Mann–Whitney *U* tests were used to compare responses between HCMV^−^ and HCMV^+^ donors, and linear regression was used to adjust for sex and age. Unless otherwise stated, statistical tests were one-sided: ****p ≤ 0.0001, ***p < 0.001, **p < 0.01, *p < 0.05.

## Results

### Donor characterization

Subject characteristics are summarized in Table I. Subjects (*n* = 152) ranged in age from 20 to 77 y (median = 33 y). Fifty-five subjects (36.2%) were found to be HCMV seropositive. Anti-HCMV IgG titer increased significantly with increasing age (*R*^2^ = 0.248, *p* = 0.0001; Supplemental Fig. 1A), but age did not differ significantly between HCMV^+^ and HCMV^−^ donors (two-tailed Mann–Whitney *U* test, *p* = 0.561). Because the proportion of female and male donors differed between the HCMV^−^ and HCMV^+^ groups, subsequent analyses were adjusted for sex.

**Table I.** Donor characteristics

	HCMV^−^ (*n* = 97)	HCMV^+^ (*n* = 55)
Median age, y (range)	32 (20–70)	35 (21–77)
Female sex, *n* (%)	73 (75)	32 (58)
*NKG2C* genotype ^+/+^, ^+/−^, ^−/−^, *n* (%)	67/24/2 (72/26/2)	35/17/2 (65/31/4)
*NKG2C*^−^ haplotype frequency (%)	15.0	19.4
Median anti-HCMV IgG titer, IU/ml (range)	<0.25	394.2 (31.1–4411.6)
Median anti-PT IgG titer, IU/ml (range)	6.7 (0.5–139.3)	5.0 (0.8–179.9)
Median anti-H1N1 IgG titer, AEUs (range)	214.6 (80.7–953.2)	190.1 (90.2–522.7)

Donors were classified as HCMV^−^ and HCMV^+^ by anti-HCMV IgG ELISA, using 0.25 IU/ml as the cutoff per manufacturer’s instructions. *NKG2* genotype (*NKG2C*^+/+^, *NKG2C*^+/−^, *NKG2C*^−/−^) was determined by PCR. IgG Ab titers against PT and H1N1 were calculated from interpolation of a reference serum or high-titer donor standard curve, respectively.

Cells from all 152 subjects were analyzed for responses to pertussis. The median anti-PT IgG titer was higher among HCMV^−^ donors than among HCMV^+^ donors, but this difference was not statistically significant (6.7 versus 5.0 IU/ml, two-tailed Mann-Whitney *U* test, *p* = 0.078). One hundred and fourteen donors (75.0%) confirmed that they had been vaccinated against pertussis, but a minority of donors reported that they had not been vaccinated against pertussis (*n* = 13; 8.6%) or were unsure of their vaccination status (*n* = 25; 16.4%). However, the proportions of these individuals did not differ between the HCMV^+^ and HCMV^−^ groups, and their Ab titers did not suggest a difference in vaccination history (data not shown).

All donors analyzed for responses to vaccine H1N1 influenza (*n* = 52) confirmed only natural exposure to H1N1, that is, no previous seasonal influenza vaccination. Median anti-H1N1 IgG titers were higher among HCMV^−^ donors (204.1 AEU/ml) than among HCMV^+^ donors (187.2 AEU/ml), although this difference was not statistically significant (two-tailed Mann–Whitney *U* test, *p* = 0.135).

### Ab and Ag-specific IL-2 drive NK cell responses to pertussis and H1N1 influenza virus

**FIGURE 1.**
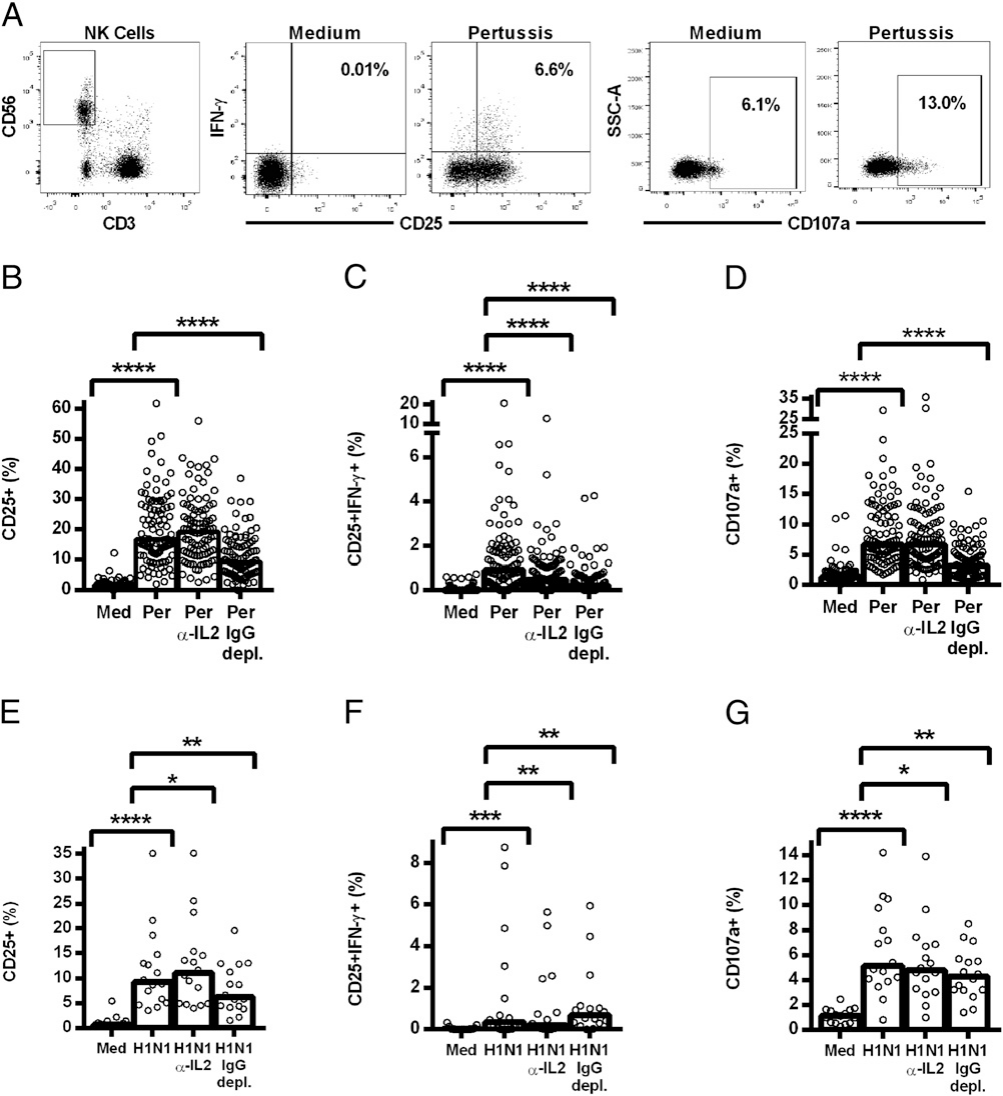
NK cell responses to pertussis and H1N1 are inhibited by IL-2 neutralization and IgG depletion. PBMCs were cultured in vitro for 18 h with medium alone, killed whole-cell pertussis (Per), and inactivated whole H1N1 influenza virus (H1N1), pertussis or H1N1 with blocking Ab to IL-2 (Per α-IL-2, H1N1 α-IL-2), or pertussis or H1N1 in IgG-depleted plasma (Per IgG depl., H1N1 IgG depl.). The isotype control Ab (IgG2A) for the IL-2 blocking Ab was included in the medium, pertussis, and H1N1 wells. Representative flow cytometry plots show gating of CD3^−^CD56^+^ NK cells and expression of CD25, IFN-γ, and CD107a (**A**). Responses to pertussis (**B**–**D**) and H1N1 (**E**–**G**) were measured by the percentage of NK cells expressing CD25 (B and E), coexpressing CD25/IFN-γ (C and F), and expressing CD107a (D and G). Data were analyzed in Prism using paired, one-tailed Wilcoxon signed-rank tests. Each data point represents one donor, *n* = 100 (B–D) or *n* = 16 (E–G), and bar graphs denote medians. ****p ≤ 0.0001, ***p < 0.001, **p < 0.01, *p < 0.05.

PBMCs from 100 donors were stimulated overnight with pertussis (Fig. 1B–D), and NK cell responses were measured by flow cytometry (Fig. 1A). Significant induction of CD25 and IFN-γ (Fig. 1B and 1C) and degranulation (CD107a; Fig. 1D) was observed in response to pertussis. Analysis of this response by CD56^bright^ and CD56^dim^ subsets reveals that the CD56^dim^ cells respond more robustly to pertussis than do the CD56^bright^ NK cells (and are thus the major contributors to the vaccine response; Supplemental Fig. 2A–C).

Coexpression of CD25/IFN-γ was markedly attenuated in the presence of a blocking Ab to IL-2 and after depletion of IgG from the plasma used to supplement the culture medium, indicating a role for both memory T cell–derived IL-2 and Ag-specific Ab in the NK cell IFN-γ response. By contrast, the degranulation response (as measured by cell-surface expression of the lysosomal marker LAMP-1/CD107a) (31) was dependent upon IgG, but not IL-2. The observation that neither anti–IL-2 nor IgG depletion completely abrogated the NK cell IFN-γ response suggests that these two signals may synergize for optimal IFN-γ production.

Cells from a subset of subjects (*n* = 16) were also analyzed for responses to H1N1 influenza in the context of IL-2 blockade or IgG depletion (Fig. 1E–G). As observed with pertussis, statistically significant induction of CD25 (Fig. 1E), CD25/IFN-γ (Fig. 1F), and CD107a (Fig. 1G) was observed in response to restimulation with H1N1 Ag, and IL-2 blocking significantly decreased CD25/IFN-γ expression (Fig. 1F), whereas IgG depletion inhibited the degranulation (CD107a) response (Fig. 1G). Interestingly, and in contrast with the response to pertussis, IgG depletion enhanced IFN-γ production in response to H1N1, and IL-2 blockade slightly decreased degranulation, indicating competition between these pathways for NK cell activation during influenza responses (Fig. 1F).

### HCMV infection is associated with impaired NK cell responses to pertussis and H1N1 influenza virus

**FIGURE 2.**
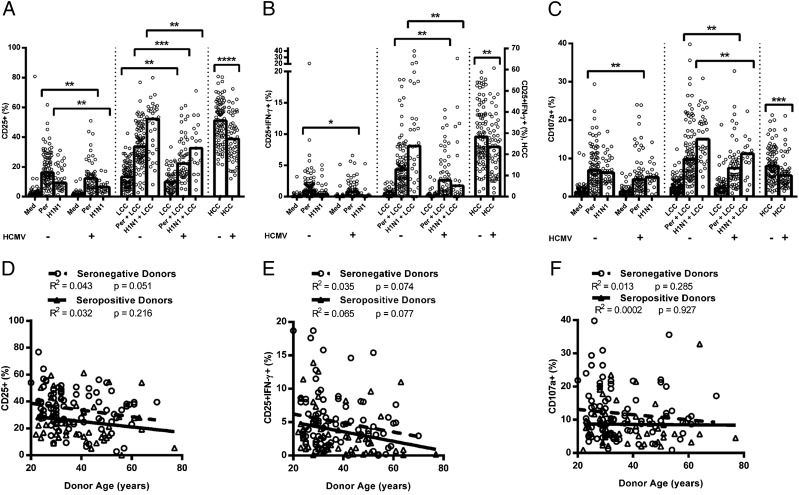
NK cell responses to vaccine Ag are affected by HCMV infection. PBMCs were cultured in vitro for 18 h with medium alone, LCC, killed whole-cell pertussis (Per), inactivated whole H1N1 influenza virus (H1N1), Per + LCC, H1N1 + LCC, or HCC. Donors were stratified into HCMV^−^ (−) and HCMV^+^ (+) groups. Responses were measured as the percentage of NK cells expressing CD25 (**A**), coexpressing CD25/IFN-γ (**B**) or CD107a (**C**). Bivariate regression of age against responses to Per + LCC was performed for the percentage of NK cells expressing CD25 (**D**), CD25/IFN-γ (**E**), and CD107a (**F**). Each data point represents one donor, *n* = 152, except for H1N1 and H1N1 + LCC where *n* = 52. Bar graphs denote medians. NB, all Ag stimulations induced statistically significant increases in expression of CD25, CD25/IFN-γ, and CD107a over background (medium alone for pertussis/H1N1, or LCC for pertussis+LCC/H1N1+LCC; *p* < 0.05 in all cases), except that H1N1 did not induce a significant increase in CD25^+^IFN-γ^+^ NK cells in HCMV^+^ donors (*p* = 0.416). Data were analyzed in Prism using, one-tailed Mann–Whitney *U* tests. ****p ≤ 0.0001, ***p < 0.001, **p < 0.01, *p < 0.05.

NK cell responses to pertussis (*n* = 152) and H1N1 (*n* = 52) were compared between HCMV^−^ and HCMV^+^ donors (Fig. 2). Consistent with prior observations (3, 6), responses to pertussis and H1N1 were significantly augmented by LCC IL-12 and IL-18 (*p* ≤ 0.0001 for all parameters), indicating that in vitro accessory cell activation and production of IL-12 and IL-18 (which is essential for IL-2–mediated NK cell activation) (3, 5, 32) were suboptimal.

Interestingly, in the absence of LCC, pertussis induces stronger NK cell responses than H1NI, whereas in the presence of LCC, H1N1 induces the most robust responses. This may indicate that pertussis induces some IL-12 and IL-18 (such that LCC is redundant in these assays), whereas H1N1 may be a poor inducer of IL-12 and IL-18 but a better inducer of IL-2 or other accessory cytokines. This would be consistent with differences in TLR signaling by RNA viruses such as influenza (TLR3) and Gram-negative bacteria such as pertussis (TLR4) (33–36).

NK cells from both HCMV^+^ and HCMV^−^ donors responded to pertussis and H1N1 (with or without LCC; Fig. 2); however, NK cell responses to these two vaccines (whether defined as CD25^+^, CD25^+^IFN-γ^+^, or CD107a^+^) were significantly lower among HCMV^+^ donors than among HCMV^−^ donors (Fig. 2A and 2B). This was true for both vaccines and all parameters when cells were cultured with LCC, and was also true for the CD25^+^ and CD25^+^IFN-γ^+^ responses to H1N1 and the CD25^+^ and CD107a^+^ responses to pertussis in the absence of LCC. Importantly, resting levels of CD25 expression did not differ significantly between HCMV^+^ and HCMV^−^ donors (Fig. 2A), and there was no difference in the potential of T cells from HCMV^−^ and HCMV^+^ donors to produce IL-2 in response to pertussis Ag (Supplemental Fig. 1B and 1C). Furthermore, there is no intrinsic difference in the ability of NK cells from HCMV^+^ and HCMV^−^ donors to degranulate in response to CD16 cross-linking or K562 stimulation (Supplemental Fig. 1D and 1E). However, NK cell CD25^+^, CD25^+^IFN-γ^+^, and CD107a^+^ expression in response to HCC (high concentrations of IL-12 and IL-18) were all significantly higher in HCMV^−^ compared with HCMV^+^ donors (Fig. 2A–C). Analysis of this response by CD56^bright^ and CD56^dim^ subsets reveals that the effect of HCMV status is due entirely to an effect within the CD56^dim^ subset (Supplemental Fig. 2D–F).

In addition to consistently lower NK cell responses to vaccine Ags in HCMV^+^ individuals, there was a trend for CD25 and CD25/IFN-γ responses to pertussis (with or without LCC) to decline with increasing age (Fig. 2D and 2E). This was statistically significant for the cohort as a whole (CD25^+^ pertussis: *R*^2^ = 0.0549, *p* = 0.0052; CD25^+^ pertussis + LCC: *R*^2^ = 0.0453, *p* = 0.0122; CD25^+^IFN-γ^+^ pertussis: *R*^2^ = 0.0379, *p* = 0.0203; CD25^+^IFN-γ^+^ pertussis + LCC: *R*^2^ = 0.0478, *p* = 0.0095), but not when analyzed separately for HCMV^−^ and HCMV^+^ donors due to decreased power. There was no effect of age on CD107a expression (pertussis: *R*^2^ = 0.00491, *p* = 0.4089; pertussis + LCC: *R*^2^ = 0.00879, *p* = 0.272; Fig. 2F), which is consistent with maturation of the NK cell repertoire, and therefore decreased sensitivity to exogenous cytokines, but maintained cytotoxicity, during normal aging (reviewed in Ref. 37) and increasing NK cell differentiation (8, 9). Importantly, the effect of HCMV infection on impaired NK cell responses to pertussis and H1N1 is entirely independent of the association between age and NK cell function. In line with this conclusion, adjusting for age by parametric regression did not alter the conclusions of the study (Table II).

**Table II.** NK cell responses to vaccine Ags by HCMV status after adjusting for sex and age

		Adjusted for Sex and Age	
Stimulus	Parameter (Total NK Cells)	Effect (95% CI)*^a^*	*p**^b^*
Pertussis	CD25^+^	−4.4 (−8.3, −0.5)	0.014
	CD25^+^IFN-γ^+^	−0.5 (−1.2, 0.3)	0.125
	CD107a^+^	−1.5 (−3.4, 0.5)	0.071
Pertussis + LCC	CD25^+^	−8.5 (−13.7, −3.4)	0.001
	CD25^+^IFN-γ^+^	−1.5 (−2.8, −0.1)	0.020
	CD107a^+^	−2.9 (−5.5, −0.3)	0.016
H1N1	CD25^+^	−5.4 (−9.5, −1.3)	0.005
	CD25^+^IFN-γ^+^	−0.4 (−1.1, 0.4)	0.158
	CD107a^+^	−1.8 (−3.9, 0.3)	0.049
H1N1 + LCC	CD25^+^	−12.2 (−22.6, −1.8)	0.011
	CD25^+^IFN-γ^+^	−5.1 (−10.4, 0.1)	0.027
	CD107a^+^	−5.1 (−8.9, −1.5)	0.004
HCC	CD25^+^	−11.3 (−16.7, −6.0)	<0.0001
	CD25^+^IFN-γ^+^	−6.5 (−11.4, −1.7)	0.005
	CD107a^+^	−2.1 (−3.5, −0.6)	0.004

A regression analysis was performed in STATA to adjust for sex and age when comparing NK cell responses to pertussis (^−/+^ LCC), H1N1 (^−/+^ LCC), and HCC between HCMV^−^ and HCMV^+^ donors. The response was quantified by the percentage of total NK cells expressing CD25, CD25/IFN-γ (CD25^+^IFN-γ^+^), and CD107a.

^*a*^ Effect (coefficient), with 95% confidence interval (CI), represents the change in the mean percentage of NK cells responding in HCMV^+^ donors as compared with HCMV^−^ donors.

^*b*^ The *p* value refers to the significance of the difference in response between HCMV^−^ and HCMV^+^ donors after adjusting for sex and age. The *p* values < 0.05 are underlined.

Overall, NK cell responses did not differ significantly between males and females, although there was a trend for median responses to be higher in women than in men, and this reached statistical significance (*p* < 0.05) for the IFN-γ response to pertussis + LCC in HCMV^+^ donors (data not shown). Because the proportion of female subjects differed between the HCMV^−^ and HCMV^+^ groups (Table I), the data in Fig. 2 were reanalyzed, adjusting for sex, as well as age, using parametric regression (Table II). After adjustment, CD25/IFN-γ and CD107a expression in response to vaccine alone (i.e., without LCC) are no longer significantly different between HCMV^−^ and HCMV^+^ donors, but responses to vaccine with LCC, and responses to HCC, remain significantly lower in HCMV^+^ compared with HCMV^−^ donors.

Finally, no associations were observed between anti-HCMV titer and any NK cell responses among the HCMV^+^ subjects, and there was no effect of *NKG2C* genotype (which may affect NK cell differentiation) (30, 38, 39) on NK cell responses (data not shown).

### NK cell differentiation only partially explains reduced responses to vaccines in HCMV^+^ donors

**FIGURE 3.**
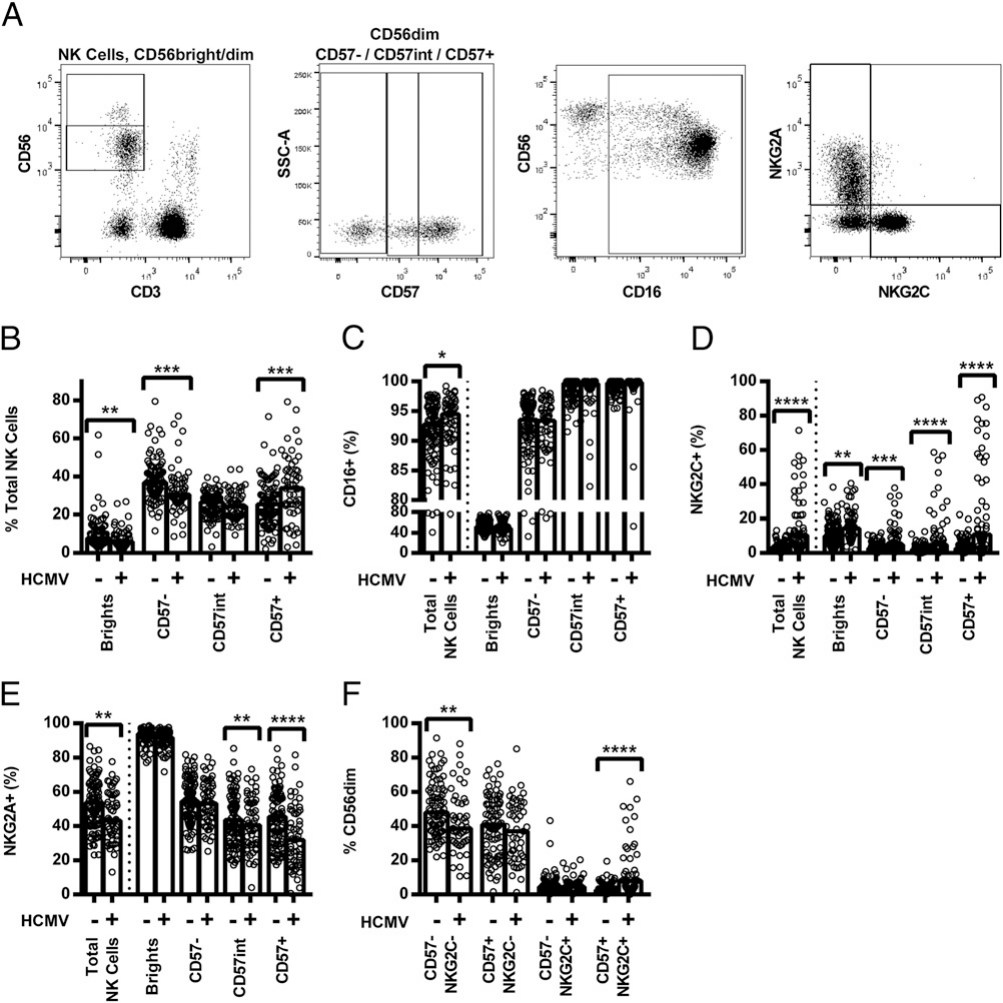
Comparison of ex vivo expression of NK cell markers and receptors in HCMV^−^ and HCMV^+^ donors. PBMCs were analyzed ex vivo for surface expression of CD56, CD57, CD16, NKG2C, and NKG2A, as shown by representative flow cytometry plots (**A**). Proportions of total NK cells in the CD56^bright^, CD56^dim^CD57^−^, CD56^dim^CD57^int^, and CD56^dim^CD57^+^ subsets were compared between HCMV^−^ and HCMV^+^ donors (**B**), as was expression of CD16 (**C**), NKG2C (**D**), NKG2A (**E**), and CD57/NKG2C (**F**, CD56^dim^ only). The percentages of cells expressing each marker in HCMV^−^ (−) and HCMV^+^ (+) donors were compared using two-tailed Mann–Whitney *U* tests. Each data point represents one donor, *n* = 152; bar graphs denote medians. ****p ≤ 0.0001, ***p < 0.001, **p < 0.01, *p < 0.05.

We hypothesized that reduced cytokine-mediated NK cell responses among HCMV^+^ donors would reflect expansion of the highly differentiated CD56^dim^CD57^+^NKG2C^+^ NK cell subset, which is known to be hyporesponsive to cytokines (12). Indeed, ex vivo analysis confirmed observations from previous studies that HCMV^+^ donors had lower proportions of CD56^dim^CD57^−^ NK cells and higher proportions of CD56^dim^CD57^+^ NK cells than did HCMV^−^ donors (Fig. 3A and 3B); there was no difference between the groups in the proportion of cells with intermediate CD57 expression (CD56^dim^CD57^int^, gating shown in Fig. 3A). Consistent with previous work (10–12, 16, 17), HCMV seropositivity was also associated with a higher proportion of CD16^+^ (Fig. 3C) and NKG2C^+^ (Fig. 3D) cells, and a lower proportion of NKG2A^+^ cells (Fig. 3E), within the total NK cell population. Moreover, HCMV seropositivity was correlated with a lower proportion of CD57^−^NKG2C^−^ cells and a higher proportion of CD57^+^NKG2C^+^ cells within the CD56^dim^ NK cell population (Fig. 3F).

**FIGURE 4.**
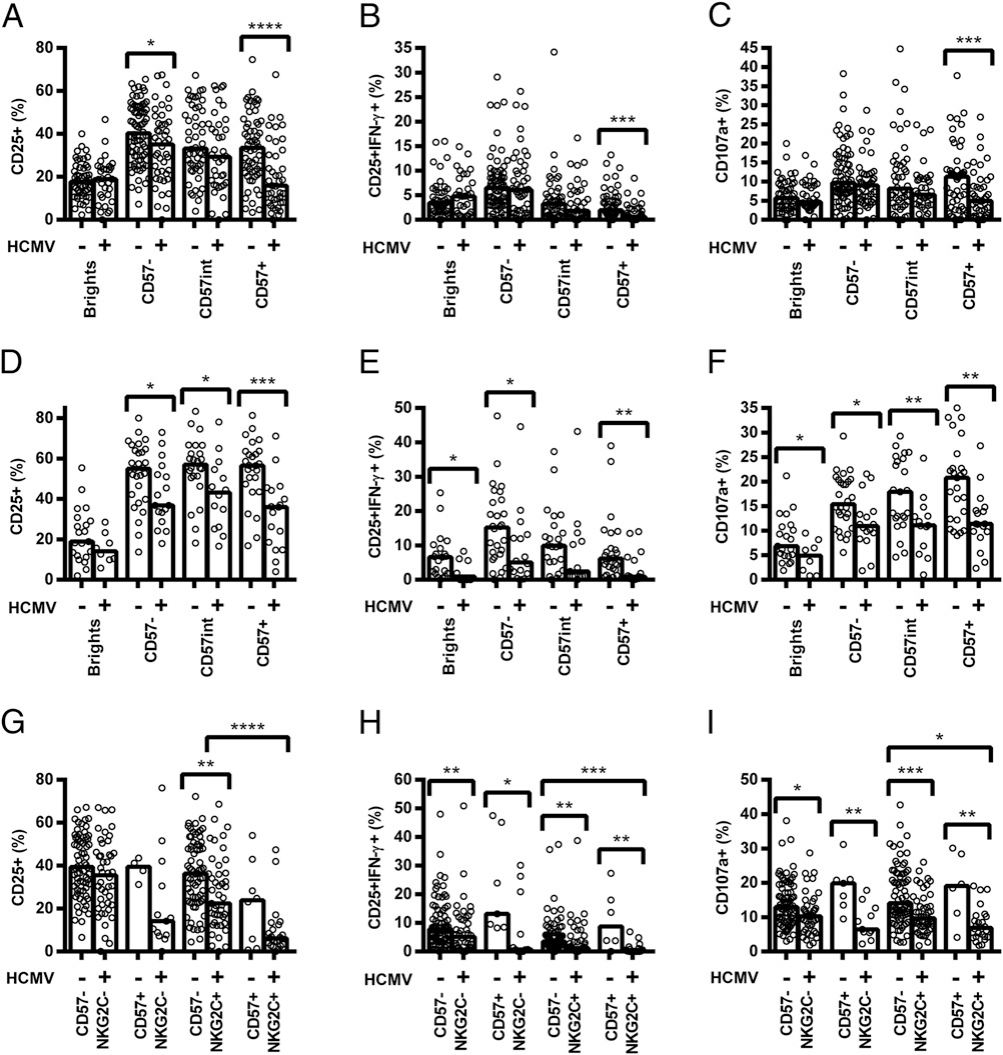
HCMV infection affects vaccine Ag responses of all NK cells, irrespective of their differentiation status. PBMCs were cultured in vitro for 18 h with killed whole-cell pertussis with LCC (pertussis + LCC) (**A**–**C** and **G**–**I**) or inactivated whole H1N1 influenza virus with LCC (H1N1 + LCC) (**D**–**F**). Responses were measured as the percentage of cells expressing CD25 (A, D, and G), CD25/IFN-γ (B, E, and H), and CD107a (C, F, and I) by CD56/CD57-defined subsets (A–F) or CD56^dim^ CD57/NKG2C-defined subsets (G–I) and compared between HCMV^−^ (−) and HCMV^+^ donors (+). Data were analyzed using one-tailed Mann–Whitney *U* tests. Each data point represents one donor, *n* = 152 (A–C and G–I) or *n* = 52 (D–F); bar graphs denote medians. NB, for CD57/NKG2C-defined subsets, CD57^int^ cells were grouped together with CD57^−^ cells. ****p ≤ 0.0001, ***p < 0.001, **p < 0.01, *p < 0.05.

Although the increased proportion of CD56^dim^CD57^+^ NK cells among HCMV^+^ donors likely contributes to their reduced responsiveness to cytokines, we also observed significantly reduced CD25, CD25/IFN-γ, and CD107a expression in response to both pertussis and H1N1 *within* individual NK cell subsets. This was especially evident among CD56^dim^CD57^+^ cells and for cultures containing LCC (Fig. 4A–F), but this was also the case for cultures stimulated with vaccine alone (Supplemental Fig. 3G–I and 3M–O).

Similarly, when cells were grouped by expression of CD57 and NKG2C, we found that responses to pertussis with LCC were lower among NKG2C^+^ NK cells than among NKG2C^−^ cells (Fig. 4G–I). This association was statistically significant for CD57^+^ NK cells of HCMV^+^ donors, but evaluation of the HCMV^−^ cohort lacked statistical power as too few donors had sufficient NKG2C^+^ cells to allow a robust analysis. Interestingly, however, responses of all four subsets were significantly lower among HCMV^+^ donors than among HCMV^−^ donors (Fig. 4G–I), despite minimal differences in responses to LCC alone (Supplemental Fig. 3A–F). These data indicate that the reduced response of HCMV^+^ donors reflects differences in the intrinsic responsiveness of NK cells within a subset, as well as differences in the distribution of these subsets. Although the level of expression (median fluorescence intensity [MFI]) of both CD57 and NKG2C was higher on CD56^dim^CD57^+^ NK cells in HCMV^+^ donors compared with HCMV^−^ donors (median MFI CD57: 13,526 versus 10,575, *p* = 0.0032; median MFI NKG2C: 141 versus 80.9, *p* < 0.0001, data not shown), there was no significant association between CD57 and NKG2C expression levels and NK cell responsiveness in HCMV^+^ donors (data not shown).

**FIGURE 5.**
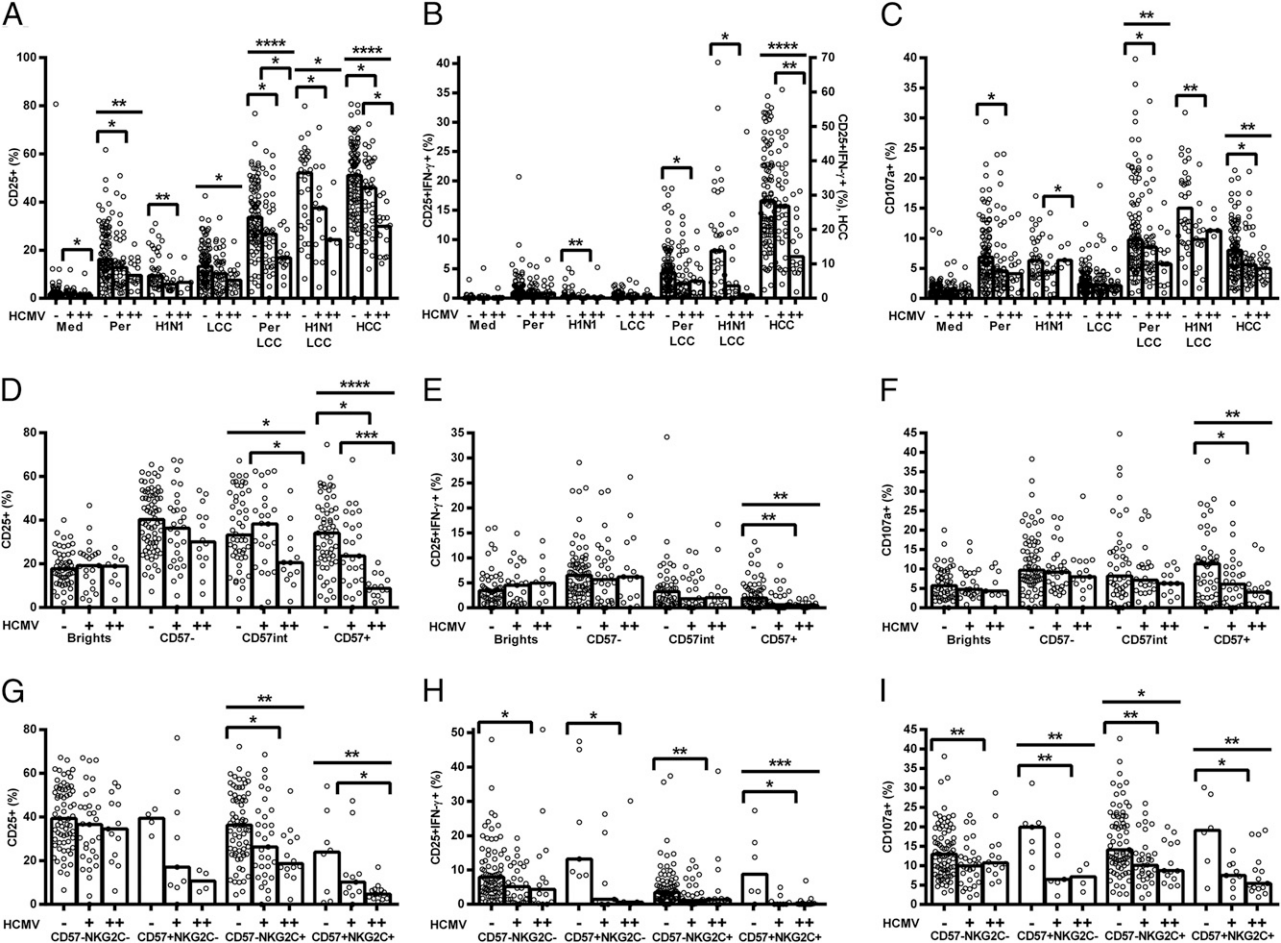
NK cell responses of HCMV^+^ donors with or without the characteristic CD56^dim^CD57^+^NKG2C^+^ expansion. PBMCs were cultured in vitro for 18 h with medium alone, LCC, killed whole-cell pertussis (Per), inactivated whole H1N1 influenza virus (H1N1), Per + LCC, H1N1 + LCC, or HCC. Donors were stratified into HCMV^−^ (−), HCMV^+^ without expansion of CD56^dim^CD57^+^NKG2C^+^ cells (+), and HCMV^+^ with expansion of CD56^dim^CD57^+^NKG2C^+^ cells (++). Responses are expressed as the percentage of total NK cells expressing CD25 (**A**), coexpressing CD25/IFN-γ (**B**), or expressing CD107a (**C**). CD57-defined (**D**–**F**) or CD57/NKG2C-defined subsets (**G**–**I**) were analyzed for responses to pertussis with LCC for CD25 (D and G), CD25/IFN-γ (E and H), and CD107a (F and I). Data were analyzed in Prism using one-tailed Mann–Whitney *U* tests to compare responses between HCMV^+^ donors and either HCMV^−^ donors or HCMV^++^ donors. ANOVA for linear trend (from − to + to ++) was also performed for each functional readout. Each data point represents one donor, *n* = 152, except for H1N1 and H1N1 + LCC where *n* = 52. Bar graphs denote medians. ****p ≤ 0.0001, ***p < 0.001, **p < 0.01, *p < 0.05.

Because only some HCMV^+^ individuals have obvious expansion of the CD56^dim^CD57^+^NKG2C^+^ subset, we considered whether NK responses might differ between HCMV^+^ individuals with and without this expanded population. Sixteen of 55 (29%) HCMV^+^ donors demonstrated expansion of the CD56^dim^CD57^+^NKG2C^+^ subset (defined as % CD56^dim^CD57^+^NKG2C^+^ cells greater than the mean + 3 SD of that in HCMV^−^ donors), and NK cells from these donors tended to respond less robustly than did cells from HCMV^+^ donors without this expansion (Fig. 5). Importantly, there was evidence by trend analysis for decreasing NK cell responsiveness with HCMV infection and then with HCMV infection plus expansion of the CD56^dim^CD57^+^NKG2C^+^ subset (Fig. 5). This confirms that although expansion of the CD56^dim^CD57^+^NKG2C^+^ subset is associated with loss of NK cell responsiveness in vaccine recall assays, cells of HCMV^+^ donors respond less well than do cells of HCMV^−^ donors, irrespective of NKG2C expression.

### HCMV infection is associated with altered expression of cytokine receptors by NK cells

Although there was a clear role for specific IgG in induction of CD25, CD25/IFN-γ, and CD107a expression (Fig. 1), impairment of CD16-mediated signaling seemed an unlikely explanation for reduced NK cell responsiveness because HCMV^+^ individuals have a higher frequency of CD16^+^ NK cells (Fig. 3C), cells from HCMV^+^ and HCMV^−^ donors responded equally well to CD16 cross-linking (Supplemental Fig. 1D), and use of pooled AB plasma for in vitro assays ensured that specific IgG concentrations were consistent in all assays.

**FIGURE 6.**
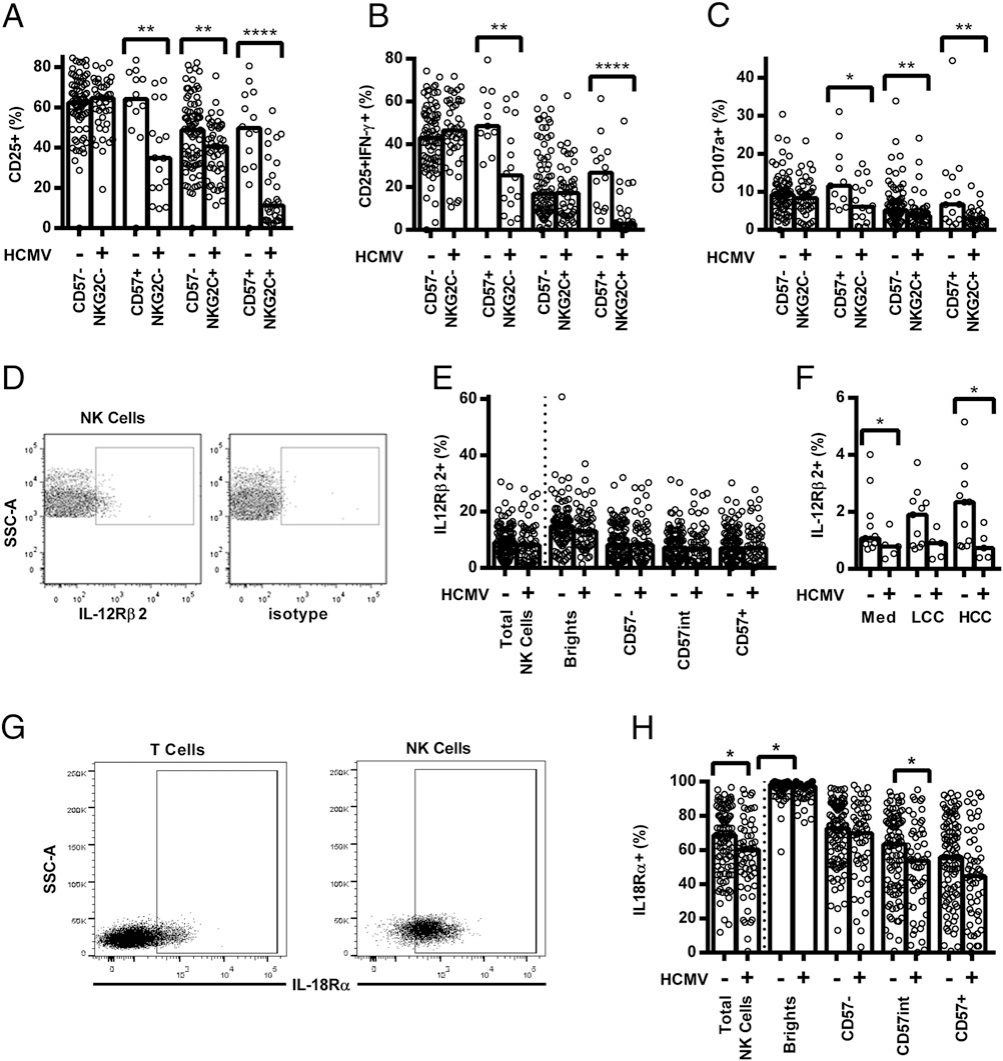
Decreased cytokine responsiveness and decreased cytokine receptor expression by NK cells from HCMV^+^ donors. (**A**–**C**) PBMCs were cultured in vitro for 18 h with an HCC. Responses were measured as the percentage of CD56^dim^ CD57/NKG2C-defined cells expressing CD25 (A), CD25/IFN-γ (B), and CD107a (C), and compared between HCMV^−^ (−) and HCMV^+^ donors (+). (**D**–**F**) NK cells were analyzed for surface expression of IL-12Rβ2 using an mIgG1 PECy5-conjugated isotype control to set the gate (D). Total NK cells (E and F) and CD56/CD57-defined subsets (E) were analyzed ex vivo (E) and after 18 h culture in vitro with LCC or HCC (F). (**G** and **H**) NK cells were also analyzed for IL-18Rα surface expression using the T cell population to set the IL-18Rα gate (G), for total NK cells and CD56/CD57-defined subsets ex vivo (H). HCMV^−^ and HCMV^+^ donors were compared using one-tailed (A–C) or two-tailed (E, F, and H) Mann–Whitney *U* tests. Each point represents one donor, *n* = 152 (A–C, E, and H) or *n* = 16 (F); bar graphs denote medians. ****p ≤ 0.0001, **p < 0.01, *p < 0.05.

In contrast, differences between HCMV^+^ and HCMV^−^ donors were most marked in cultures containing LCC (Fig. 2) and in cultures with high concentrations of the cytokines IL-12 and IL-18 (HCC; Fig. 6A–C), suggesting that differences in expression of cytokine receptors might explain our observations. Although there was no difference in resting (ex vivo) expression of IL-12Rβ2 on any NK cell subset (Fig. 6D and 6E), IL-12Rβ2 was significantly upregulated on the total NK cell population in HCMV^−^ but not from HCMV^+^ donors after culture with HCC (Fig. 6F). Moreover, and consistent with data showing associations between acquisition of CD57 and decreased IL-18Rα expression (6, 8, 9), resting NK cells from HCMV^+^ donors were significantly less likely than cells from HCMV^−^ donors to express IL-18Rα, and this difference was especially marked in the (expanded) CD56^dim^CD57^+^ NK cell subset (Fig. 6G and 6H).

## Discussion

During secondary immune responses, both CD4^+^ T cell–derived IL-2 and Ag–Ab immune complexes induce “Ag-specific” NK cell activation, allowing NK cells to act as effectors of the adaptive immune response and to contribute to postvaccination immunity by secretion of IFN-γ and/or by cytotoxicity (3–6, 14). In this study, we demonstrate for the first time, to our knowledge, that the contribution of NK cells to adaptive immune responses is affected by HCMV infection: NK cells from HCMV^+^ donors respond significantly less well than cells from HCMV^−^ donors to killed whole-cell pertussis or inactivated whole H1N1 influenza virus. The effect of HCMV infection on NK cell responsiveness is independent of age, sex, or anti-HCMV IgG titer.

Our data also demonstrate for the first time, to our knowledge, that there is an additive effect between the cytokine and the IgG pathways driving NK cell IFN-γ production, because both IgG depletion and IL-2 blockade reduced NK cell IFN-γ responses in response to stimulation of PBMCs with pertussis vaccine. Of particular interest, IgG depletion markedly reduced Ag-induced CD25 expression on NK cells. We propose that CD16 cross-linking by immune complexes upregulates CD25 expression, increasing sensitivity to T cell–derived IL-2, and thereby enhancing IFN-γ production. However, CD16 cross-linking is not essential for upregulation of CD25, because this can be induced by Ag alone, presumably in response to IL-12 and IL-18 produced by APCs (6, 40–42). Release of cytotoxic granules, as measured by upregulation of CD107a on the cell surface, is also inhibited by IgG depletion but is unaffected by IL-2 blockade, suggesting that NK cells could act as effectors of the adaptive response through ADCC in the absence of memory T cells, providing there is sufficient circulating Ab.

However, whereas IgG depletion also decreased H1N1-induced CD25 expression on NK cells, H1N1 induction of IFN-γ was significantly enhanced in the absence of IgG. We have observed that individual NK cells tend to either produce IFN-γ or degranulate (but not both; unpublished data), suggesting that inhibiting the degranulation response to H1N1 by removing IgG skews the response toward IFN-γ production. However, given the limited effect of IgG depletion on H1N1-induced degranulation, it is unclear why this should be the case. Indeed, expression of CD107a in response to H1N1 seems to be relatively unaffected by either IL-2 blockade or IgG depletion. This suggests that H1N1-driven degranulation may be affected by other stimuli, such as type I IFNs (43, 44).

We had hypothesized that decreased responses to vaccines in HCMV^+^ donors would be attributable to a redistribution of the NK cell repertoire. HCMV infection drives the expansion of a CD56^dim^CD57^+^NKG2C^+^ subset of NK cells (11, 16, 17, 45), which display a highly differentiated phenotype, including reduced responsiveness to exogenous cytokine stimulation (8, 9) and epigenetic changes at the *IFNG* locus (46). These phenotypic and functional changes are similar to those observed during aging (15, 47), and comparisons have been drawn between the effects of HCMV and immunosenescence (48). Because our previous work has indicated that NK cell IFN-γ production after restimulation with vaccine Ags is cytokine dependent (3), we predicted that fewer NK cells from HCMV^+^ donors would produce IFN-γ in response to pertussis or influenza Ags because of the reduced capacity of the expanded CD56^dim^CD57^+^NKG2C^+^ subset to respond to cytokines. Ex vivo analyses confirmed that HCMV^+^ donors had higher proportions of CD56^dim^CD57^+^ and CD56^dim^CD57^+^NKG2C^+^ NK cells than did HCMV^−^ donors, and functional analysis confirmed that few of the highly differentiated CD57^+^ NK cells produced IFN-γ after Ag stimulation. Interestingly, however, our data also show that, irrespective of their CD57/NKG2C surface phenotype, NK cells from HCMV^+^ donors are less likely to produce IFN-γ in response to vaccines than are cells from HCMV^−^ donors. In other words, there are pronounced functional differences between HCMV^+^ and HCMV^−^ donors *within* NK cell subsets. The reduced NK cell IFN-γ response to vaccine Ags in HCMV^+^ donors is therefore not simply due to expansion of the CD56^dim^CD57^+^NKG2C^+^ subset. Although acquisition of NKG2C was functionally relevant (associated with reduced IFN-γ and degranulation responses), it was not sufficient to explain the reduced responsiveness of cells from HCMV^+^ donors.

Although further studies are required to define the “within subset” effects of HCMV infection, our data suggest that reduced expression of IL-18Rα or reduced ability to upregulate IL-12Rβ2 among NK cells from HCMV-infected individuals may partially explain their failure to produce IFN-γ. Although decreasing expression of IL-12Rβ2 and IL-18Rα has been associated with CD57 expression, this is the first demonstration, to our knowledge, that there are differences in cytokine receptor expression between HCMV^+^ and HCMV^−^ donors and it is possible to see how each of these might affect NK cell responses. Higher resting levels of IL-18Rα expression would increase the sensitivity of NK cells to low concentrations of IL-18 being produced by APCs in response to innate receptor ligands in whole-cell pertussis or inactivated influenza virus. IL-18 signaling upregulates CD25 (49), thereby increasing sensitivity to IL-2. IL-2 signaling might then upregulate IL-12R2β (50, 51), allowing IL-12 to synergize with IL-2 to drive IFN-γ production (3, 40, 52), while also generating a positive feedback loop in which IL-12 signaling upregulates IL-18Rα (53, 54), IL-18 signaling, and CD25. However, although cytokine receptor expression is likely to play a role in determining NK cell responsiveness to vaccine Ags in HCMV^−^ and HCMV^+^ donors, the biological relevance of small changes in surface expression on IL-12Rβ2 needs to be demonstrated. Moreover, although we have no evidence to suggest that T cell IL-2 production in response to vaccine Ags is affected by HCMV infection, future studies will need to determine the extent to which concomitant changes in APC function during HCMV infection also affect NK cell responses.

We had initially considered NK cell degranulation during vaccine restimulation to be a result of CD16 cross-linking by IgG immune complexes, as suggested by the IgG depletion data and accepted models of ADCC. The expectation was, therefore, that although IFN-γ responses might be impaired, NK cell degranulation responses would be sustained in HCMV^+^ donors. Indeed, cross-linking with anti-CD16 Ab induced equivalent levels of CD107a upregulation. It was, therefore, somewhat surprising that degranulation responses to vaccine were lower in HCMV^+^ donors than in HCMV^−^ donors. However, degranulation responses to HCC were also lower in HCMV^+^ donors, supporting the notion of synergy between the cytokine and CD16 pathways, and adding weight to the suggestion that HCMV infection may affect cytokine receptor expression.

Our findings have potentially important implications. HCMV infection is a known risk factor for all-cause mortality in adults (55), and perinatal HCMV infection is associated with slower growth and increased rates of hospitalization in African children (56). The underlying biology of these relationships is unknown, but reduced responsiveness to vaccination or reduced resilience in the face of infection are plausible explanations. Distorted T cell and NK cell phenotypes in HCMV^+^ individuals have been widely reported (15, 57–59), giving credence to the possibility that adaptive immune responses may be less effective in infected individuals. Further work will need to address the clinical consequences of altered NK cell responses to infection and vaccination in HCMV-infected individuals.

To our knowledge, this is the first published study of the effect of HCMV infection on NK cell responses to vaccine Ags. When compared with the marked effect of HCMV on cellular immune responses in our adult cohort, the modest phenotype seen in the infant studies (24, 25) raises the intriguing question whether the duration of HCMV infection affects vaccine responses. We have previously shown in an African population that, with near-universal infant HCMV infection, the characteristic “adult HCMV” NK cell profile is reached by early adolescence (30). The majority of our donors are of European or North American origin (data not shown), suggesting that they may have been infected in adolescence or adulthood (60, 61), potentially explaining some of the heterogeneity in the responses we see within the HCMV^+^ group. Similarly, there will be variation among our donors in time since vaccination (pertussis) or infection (H1N1), and it is likely that relatively low IFN-γ responses we observe in comparison with earlier studies (3) is due to the much longer interval between primary and secondary exposures to Ag. Future studies will need to assess whether the duration of HCMV infection is a risk factor for altered NK responses and whether this manifests itself as reduced responsiveness to active vaccination and reduced vaccine efficacy.

## Supplementary Material

Data Supplement
