# Supplementary material for: Impaired NK Cell Responses to Pertussis and H1N1 Influenza Vaccine Antigens in Human Cytomegalovirus-Infected Individuals
Source: J Immunol. 2015 Apr 8;194(10):4657–67. doi: 10.4049/jimmunol.1403080 (PMC4416741; doi:10.4049/jimmunol.1403080)
Supplement: Data Supplement [file JI_1403080.zip › JI_1403080_Supplemental_Figures_1.pdf]

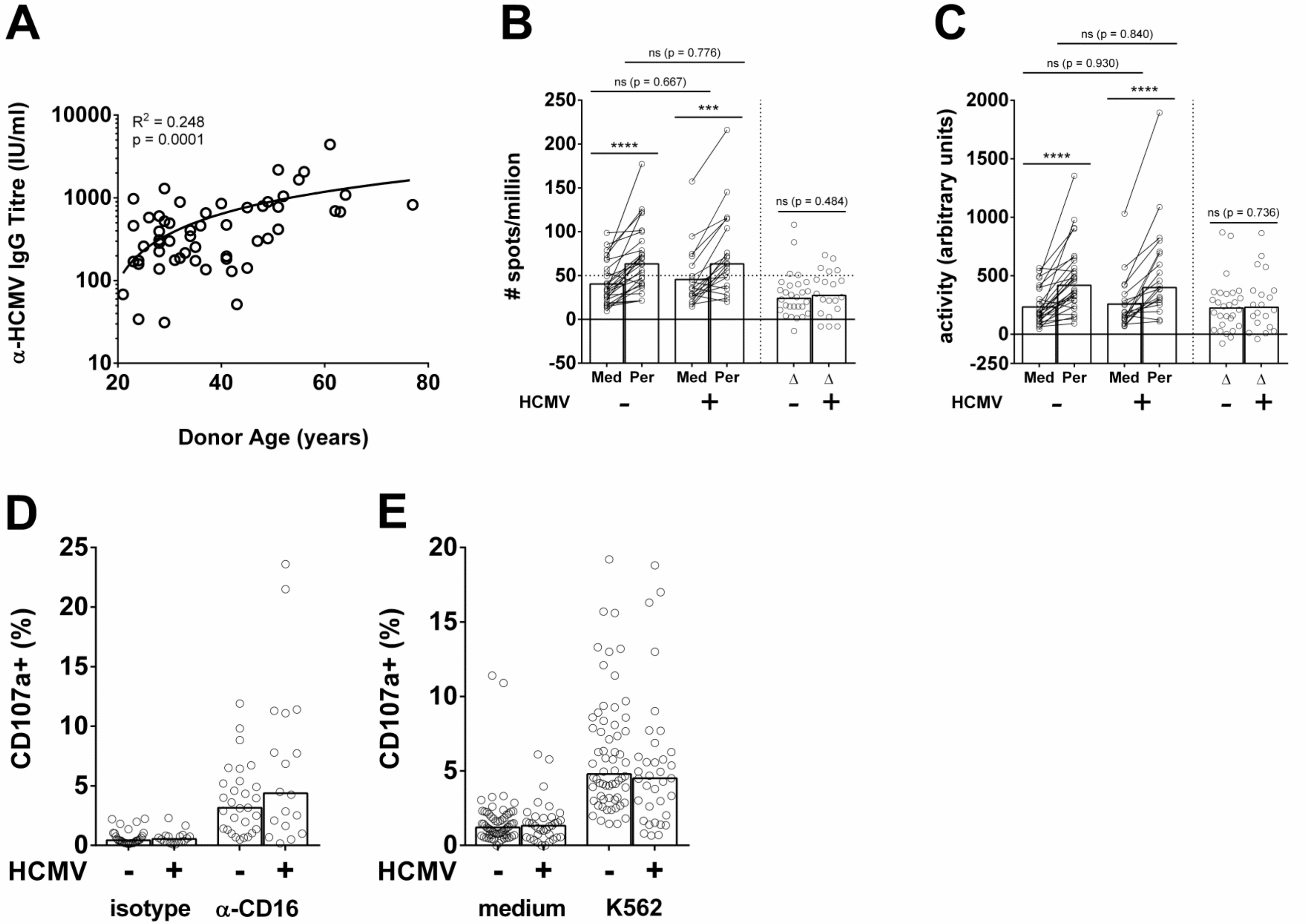

Supplemental Figure 1.

(A) Anti-human cytomegalovirus (HCMV) IgG titres increase with age in HCMV seropositive donors. Bivariate nonlinear regression of donor age against anti-HCMV IgG titre in HCMV seropositive donors was performed in Prism. Each data point represents one donor, *n* = 55.

(B-C) IL-2 production in response to pertussis is equally robust in human cytomegalovirus (HCMV) seronegative and HCMV seropositive donors. PBMC were cultured *in vitro* for 18hrs with killed whole cell pertussis (Per) or without (Med; medium) and assessed for IL-2 production using a human IL-2 ELISPOT (ALP) kit (Mabtech), as per manufacturer's instructions. Responses were measured in terms of the number of cells producing IL-2 (spots/million, B) and the amount of IL-2 produced (activity, C). Delta (Δ) values were calculated by subtracting the background response (Med) from the response to pertussis (Per). Responses to pertussis for each donor were compared to medium alone using two-tailed Wilcoxon tests, and responses were compared between HCMV seronegative (-) and HCMV seropositive (+) donors using two-tailed Mann-Whitney tests. **** $p \leq 0.0001$, *** $p < 0.001$. Each data point represents the mean of three technical replicates for a single donor (*n* = 48) and bar graphs denote medians.

(D-E) Degranulation responses after CD16 crosslinking or K562 stimulation are equally robust in human cytomegalovirus (HCMV) seronegative and HCMV seropositive donors. PBMC were cultured *in vitro* for 5hr in a 96-well flat-bottomed plate coated with IgG1 isotype control (isotype) or anti-CD16 antibody (D), or for 18hr in a 96-well round-bottomed plate with medium alone or K562 cells, at an effector:target (E:T) ratio of 2:1 (E). Responses were measured as the percentage of NK cells expressing CD107a and were compared between HCMV seronegative (-) and HCMV seropositive donors (+) using two-tailed Mann-Whitney tests (ns). Each data point represents one donor, *n* = 52 (D) or *n* = 100 (E), and bar graphs denote medians.

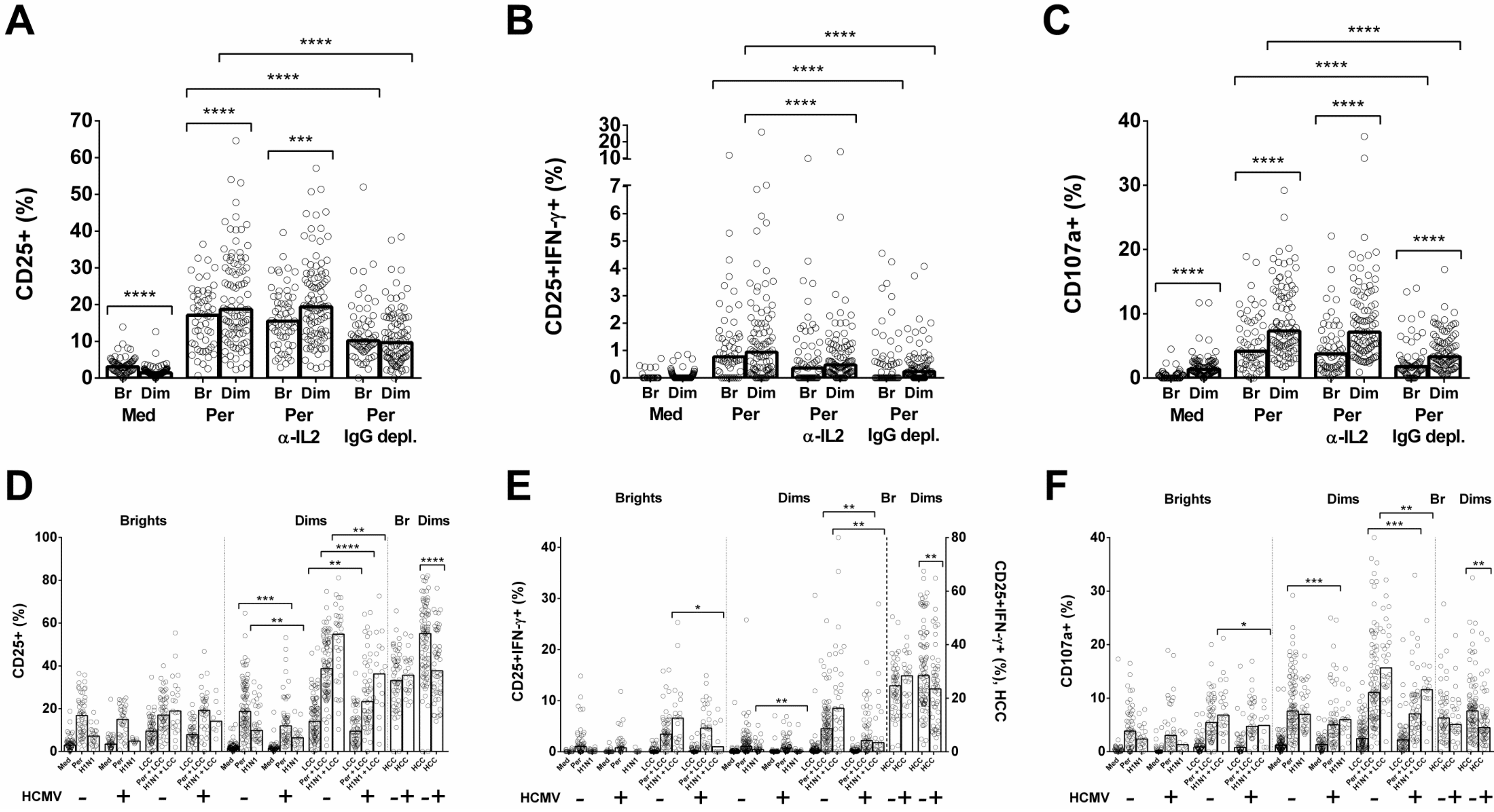

Supplemental Figure 2.

(A-C) CD56bright and CD56dim natural killer (NK) cell responses are inhibited by IL-2 neutralisation and IgG depletion. PBMC were cultured *in vitro* for 18hr with medium alone, killed whole cell pertussis (Per), pertussis with blocking antibody to IL-2 (Per α-IL2) or pertussis in IgG-depleted plasma (Per IgG depl.). The isotype control antibody (IgG2A) for the IL-2 blocking antibody was included in the medium and pertussis wells. Responses were measured by the percentage of CD56bright (Br) or CD56dim (Dim) NK cells expressing CD25 (A), co-expressing CD25/IFN-γ (B), and expressing CD107a (C). CD56bright and CD56dim responses were compared to each other for each condition, then to pertussis alone for the IL-2 blocking and IgG depletion conditions. Data were analysed in Prism using paired, one-tailed Wilcoxon signed-rank tests. **** $p \leq 0.0001$, *** $p < 0.001$.. Each data point represents one donor, $n = 100$, and bar graphs denote medians.

(D-F) CD56bright and CD56dim natural killer (NK) cells respond more poorly to vaccine antigens in HCMV seropositive donors as compared to HCMV seronegative donors. PBMC were cultured *in vitro* for 18hr with medium alone, low concentration of cytokines (LCC), killed whole cell pertussis (Per), inactivated whole H1N1 influenza virus (H1N1), Per + LCC, H1N1 + LCC, or high concentration of cytokines (HCC). Donors were stratified into HCMV seronegative (-) and HCMV seropositive (+) groups. Responses were measured as the percentage of CD56bright or CD56dim NK cells expressing CD25 (D), co-expressing CD25/IFN-γ (E), or CD107a (F). Data were analysed in Prism using, one-tailed Mann-Whitney tests. **** $p \leq 0.0001$, *** $p < 0.001$, ** $p < 0.01$, * $p < 0.05$. Each data point represents one donor, $n = 152$, except for H1N1 and H1N1 + LCC where $n = 52$. Bar graphs denote medians.

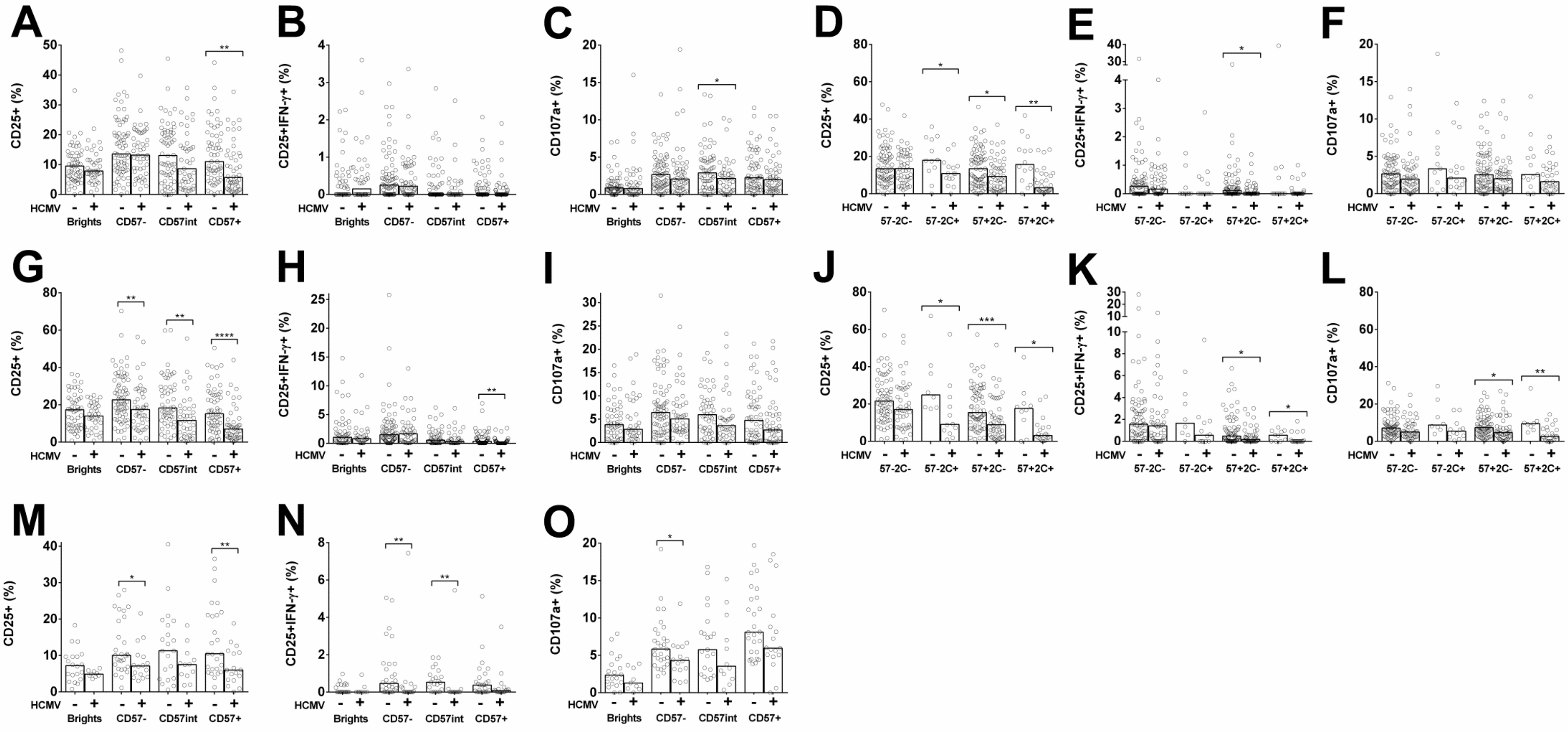

Supplemental Figure 3. Natural killer (NK) cell subset responses to LCC or vaccine antigen alone by human cytomegalovirus (HCMV) status. PBMC were cultured *in vitro* for 18hr with a low concentration of cytokines (LCC; A-F), killed whole cell pertussis (G-L) or inactivated whole H1N1 influenza virus (M-O). Responses were measured as the percentage of CD57-defined (A-C, G-I, M-O) or CD57/NKG2C-defined (D-F, J-L) NK cells expressing CD25 (A, D, G, J, M), CD25/IFN-γ (B, E, H, K, N), and CD107a (C, F, I, L, O) and were compared between HCMV seronegative (-) and HCMV seropositive donors (+). Data were analysed using one-tailed Mann-Whitney tests. **** $p \leq 0.0001$, *** $p < 0.001$, ** $p < 0.01$, * $p < 0.05$. Each data point represents one donor, n = 152 (A-L) or n = 52 (M-O), and bar graphs denote medians.
